# Boosting the vascularization and regenerative capacity of nanofat by short-term ex vivo pretreatment with erythropoietin

**DOI:** 10.1186/s12967-026-08640-x

**Published:** 2026-07-13

**Authors:** Valeria Pruzzo, Francesca Bonomi, Ettore Limido, Andrea Weinzierl, Yves Harder, Matthias W. Laschke

**Affiliations:** 1 Institute for Clinical and Experimental Surgery, Saarland University, PharmaScienceHub (PSH), 66421 Homburg, Germany; 2 Department of Surgery, Ospedale Beata Vergine Mendrisio, Ente Ospedaliero Cantonale (EOC), Mendrisio, 6850 Switzerland; 3 Department of Plastic Surgery and Hand Surgery, University Hospital Zurich, Zurich, 8006 Switzerland; 4 Department of Plastic, Reconstructive, and Aesthetic Surgery and Hand Surgery, Centre Hospitalier Universitaire Vaudois (CHUV), Lausanne, 1011 Switzerland; 5 Faculty of Biology and Medicine, University of Lausanne (UNIL), Lausanne, 1011 Switzerland

**Keywords:** Erythropoietin, Nanofat, Dermal Substitute, Vascularization, Angiogenesis, Inflammation, Biocompatibility

## Abstract

**Background:**

Erythropoietin (EPO) is a glycoprotein hormone that exerts pro-angiogenic and anti-inflammatory effects. The present study investigated whether this beneficial profile of action is suitable for improving the in vivo performance of nanofat, an emulsified fat derivative that is clinically used in plastic and reconstructive surgery.

**Results:**

Repeated intravital fluorescent microscopic analyses showed that EPO-pretreated nanofat significantly accelerates and enhances the vascularization of the implants, as evidenced by an earlier onset of blood perfusion and an increased functional microvessel density when compared to controls. This was associated with a reduced inflammatory response to the implants, as indicated by lower numbers of adherent leukocytes in venules of the host tissue. Histological and immunohistochemical analyses further revealed an improved implant integration with an increased collagen I deposition and a higher density of nanofat-derived CD31⁺/green fluorescent protein (GFP^+^) microvessels, along with a reduced macrophage and neutrophil infiltration.

**Methods:**

Nanofat was mechanically generated from subcutaneous adipose tissue of GFP^+^ C57BL/6J mice and incubated for 1 h in Hank’s Balanced Salt Solution with or without EPO (3 IU/mL). The pretreated nanofat was seeded onto dermal substitutes, which were implanted into dorsal skinfold chambers of GFP⁻ C57BL/6J mice and analyzed over 14 days.

**Conclusion:**

These findings identify short-term pretreatment with EPO as an effective strategy to boost the vascularization and regenerative capacity of nanofat.

## Background

Erythropoietin (EPO) is a glycoprotein hormone that, beyond its hematopoietic function, exerts broad tissue-protective and regenerative effects, including the stimulation of angiogenesis as well as the suppression of apoptosis and inflammation [1, 3]. Accordingly, the administration of EPO has been proven to be a promising approach in preclinical models of wound healing, flap surgery, peripheral nerve regeneration, fat grafting and bone repair [4]. Moreover, EPO has also been used to increase the efficacy of other therapeutic strategies, such as the transplantation of adipose tissue-derived microvascular fragments (MVFs) as vascularization units for implanted tissue-engineered constructs. In fact, Karschnia et al. [5] reported that a 24-hour ex vivo cultivation of these enzymatically isolated vessel segments from fat samples in EPO-supplemented medium significantly increases the proliferation of their endothelial cells and inhibits apoptotic cell death of their perivascular cells. Consequently, dermal substitutes seeded with EPO-pretreated MVFs exhibited a markedly improved vascularization after implantation into recipient mice [5].

Nanofat is another adipose tissue derivative already in clinical use for the treatment of chronic wounds and scars as well as for facial rejuvenation [6, 9]. In contrast to MVFs, nanofat can be generated without collagenase digestion by simple mechanical emulsification and filtration of adipose tissue, which facilitates its clinical translation due to a lower risk for patients and, thus, no major ethical and regulatory hurdles [10, 12]. Of note, nanofat also contains MVFs, but also adipose-derived stem cells (ASCs), extracellular matrix components and angiogenic growth factors, all of which contribute to its regenerative activity [13, 14]. Hence, nanofat-seeded dermal substitutes showed an improved vascularization and tissue integration after in vivo implantation [15]. However, during the first days, these implants still lacked adequate blood perfusion, which is a mandatory prerequisite for their subsequent coverage with split-skin grafts in the clinical therapy of full-thickness skin defects following burns, traumatic injuries and oncologic resections [16]. Therefore, we have recently started to evaluate novel approaches for further boosting the regenerative properties of nanofat. In a first study, we demonstrated that this can be achieved to a limited extent by incubating isolated nanofat for 1 h in Hank’s Balanced Salt Solution (HBSS) supplemented with highly concentrated glucose (30 mM) [17]. Accordingly, this first study provided the proof-of-concept that short-term ex vivo pretreatment of nanofat is a valid strategy to improve its subsequent in vivo therapeutic performance.

Based on the promising and broad application spectrum of EPO in plastic and reconstructive surgery and our own preliminary results on the ex vivo activation of nanofat, we analyzed in the present study, whether short-term exposure of nanofat to EPO (3 IU/mL) may effectively enhance its vascularization and regenerative capacity. For this purpose, EPO-pretreated and non-pretreated control nanofat was seeded onto dermal substitutes, which were analyzed in a well-established mouse dorsal skinfold chamber model by means of intravital fluorescence microscopy, histology and immunohistochemistry throughout an observation period of 2 weeks. Accordingly, it was possible to study the vascularization of the implants during the most critical initial phase after their implantation, which determines their barrier function and suitability for subsequent split-skin grafting.

## Methods

### Animals

**Fig. 1:**
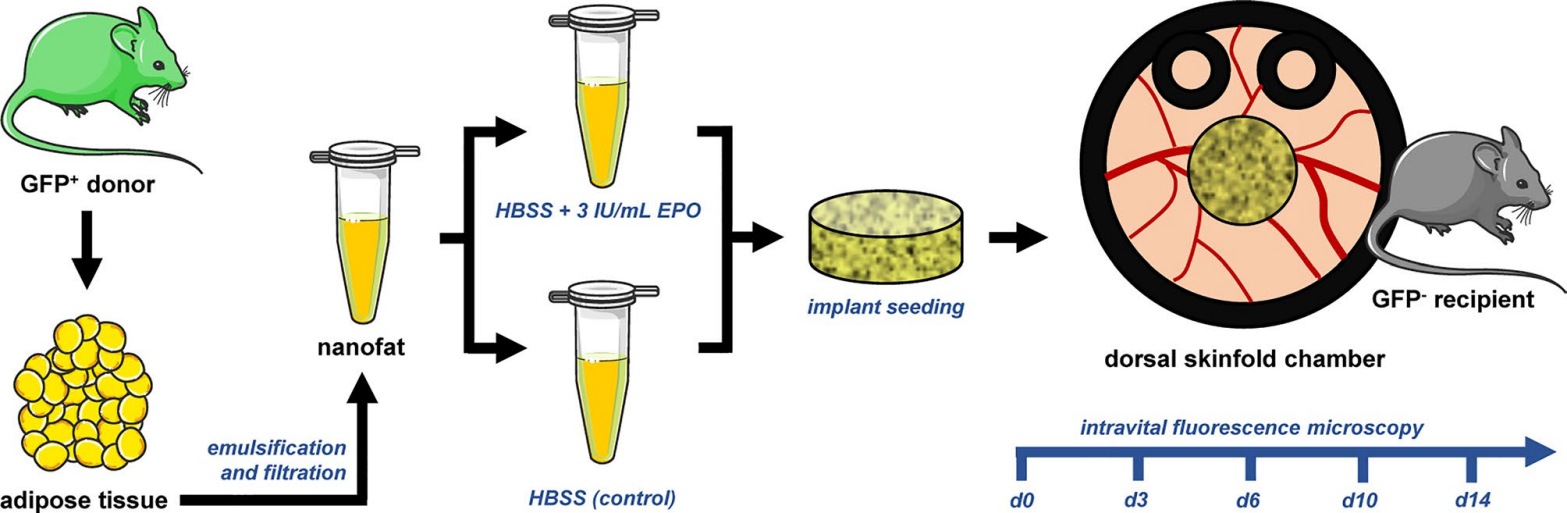
Experimental protocol of the present study. Subcutaneous adipose tissue was harvested from GFP^+^ C57BL/6J donor mice and further processed by mechanical emulsification and filtration into nanofat, which was incubated for 1 h in HBSS with or without EPO (3 IU/mL). The pretreated nanofat was then seeded onto dermal substitutes, which were implanted into dorsal skinfold chambers of GFP⁻ C57BL/6J recipient mice and repeatedly analyzed over 14 days by means of intravital fluorescence microscopy. Thereafter, the implants and their surrounding tissue were additionally examined by histology and immunohistochemistry

Inguinal fat pads were harvested from 8 male and female green fluorescent protein (GFP)^+^ C57BL/6-Tg (CAG-EGFP)131Osb/LeySopJ mice (age: ~9 months; weight: >30 g; The Jackson Laboratory, Bar Harbor, ME, USA) to collect enough adipose tissue for nanofat preparation (Fig. 1). Both male and female donors could be used because donor sex does not markedly affect the in vivo vascularization capacity of nanofat [13]. Additionally, dorsal skinfold chambers were implanted in 16 male and female GFP^−^ C57BL/6J wild-type mice (age: ~5 months; weight: ~22–30 g). These mice were housed on wood chips as bedding under standard laboratory conditions (temperature: 22 ± 2 °C; humidity: 55 ± 10%) with free access to water and pellet chow (ssniff Spezialdiäten GmbH, Soest, Germany). The animals were either bred at the Institute for Clinical and Experimental Surgery (Saarland University, Homburg, Germany) or obtained from Charles River Laboratories (Sulzfeld, Germany).

All animal procedures were approved by the local authorities (permission number: 19-2024; State Office for Consumer Protection, Saarbrücken, Germany). The experiments were conducted in accordance with the European Directive 2010/63/EU on the protection of animals used for scientific purposes, the ARRIVE Guidelines and the National Institutes of Health (NIH) Guidelines for the Care and Use of Laboratory Animals (NIH publication #85 − 23 Rev. 1985).

### Anesthesia

For all surgical procedures and microscopic analyses, the mice were anesthetized by intraperitoneal injection of ketamine hydrochloride (100 mg/kg body weight; Ketabel^®^, Bela-pharm GmbH & Co. KG, Vechta, Germany) in combination with xylazine (12 mg/kg body weight; Rompun^®^, Bayer, Leverkusen, Germany). Perioperative analgesia was provided by subcutaneous administration of carprofen (10 mg/kg body weight; Rimadyl^®^, Zoetis Deutschland GmbH, Berlin, Germany). To prevent corneal drying during anesthesia, an ophthalmic ointment (Bepanthen^®^, Bayer Vital GmbH, Leverkusen, Germany) was applied.

### Preparation and pretreatment of nanofat

The white subcutaneous adipose tissue harvested from GFP^+^ donor mice was mechanically processed into nanofat following established protocols [6, 15]. Briefly, the tissue was washed in physiological saline and minced into small fragments (~ 1 mm × 1 mm × 1 mm) using a McIlwain Tissue Chopper (CLE Co. Ltd., Gomshall, UK). These fragments were then repeatedly shuffled between two syringes through a series of three female-to-female Luer lock reducers with diameters of 2.4, 1.4 and 1.2 mm (30 cycles per connector). The resulting suspension underwent a final filtration step through a 500 μm filter to remove larger tissue remnants. The processed nanofat was then divided equally into two Eppendorf tubes (Eppendorf, Hamburg, Germany) and incubated for 1 h in a 1:1 volume ratio with either Hank’s Balanced Salt Solution (Gibco, Waltham, MA, USA) alone (vehicle control; *n* = 8) or HBSS supplemented with 3 IU/mL of EPO beta (*n* = 8; NeoRecormon^®^, Roche, Basel, Switzerland) (Fig. 1).

### Implant seeding with pretreated nanofat

The dermal substitute Integra^®^ (Integra LifeSciences, Ghent, Belgium) with a thickness of 1.3 mm was used to punch out 4-mm circular discs using a sterile tissue punch (Kai Europe GmbH, Solingen, Germany). The discs were subsequently transferred to tubes containing either vehicle-pretreated (control) or EPO-pretreated nanofat and incubated at room temperature for 10 min. This brief incubation step ensured that the pretreated nanofat adhered to the implants’ porous structure, a process previously verified by histological assessment [15].

### Dorsal skinfold chamber model and microscopic analysis

Dermal substitutes seeded with either vehicle- or EPO-pretreated nanofat were implanted into the observation window of dorsal skinfold chambers in GFP⁻ recipient mice. This approach allowed longitudinal intravital fluorescence microscopy of the implants on days 0 (implantation), 3, 6, 10 and 14 (Fig. 1). For plasma and leukocyte visualization, mice received retrobulbar intravenous injections of 50 µL of 5% fluorescein isothiocyanate (FITC)-labeled dextran (150,000 Da; Sigma-Aldrich, Taufkirchen, Germany) and 50 µL of 0.1% rhodamine 6G (Sigma-Aldrich). Imaging was performed using a Zeiss Axiotech fluorescence epi-illumination microscope (Zeiss, Oberkochen, Germany) equipped with an Axiocam 702 mono camera (Carl Zeiss Microscopy GmbH, Oberkochen, Germany). Image processing and quantitative analyses were conducted with the CapImage system (version 8.10.1; Dr. Zeintl Software, Heidelberg, Germany).

Eight regions of interest (ROIs) located at the border (*n* = 4) and center (*n* = 4) of the implants were examined. Newly formed red blood cell (RBC)-perfused microvessels were identified to assess the number of perfused ROIs (expressed as % of all ROIs). The total length of RBC-perfused microvessels per ROI was measured to calculate the functional microvessel density (expressed as cm/cm^2^). Additionally, 5 microvessels within each ROI were randomly selected to measure the vessel diameter (µm) and the centerline RBC velocity (µm/s). These values were subsequently used to compute shear rate (s⁻¹) and volumetric blood flow (pL/s) [18].

The inflammatory response to the implants was assessed in four peri-implant postcapillary and collecting venules by analyzing microhemodynamic parameters (vessel diameter, centerline RBC velocity, shear rate and volumetric blood flow) and leukocyte-endothelial cell interactions. Leukocytes were classified as free-flowing, rolling or adherent. Rolling leukocytes (min⁻¹) were defined by their reduced velocity and intermittent contact with the endothelial surface. In contrast, adherent leukocytes (mm⁻^2^) were characterized by firm attachment to the endothelium for at least 30 s. The endothelial surface area was calculated assuming a cylindrical vessel geometry.

### Histology and immunohistochemistry

After completion of the final intravital microscopy, the mice were euthanized by an overdose of anesthesia followed by cervical dislocation. The dorsal skin containing the implanted dermal substitutes was carefully excised and processed for histological and immunohistochemical stainings. Hematoxylin and eosin (HE) staining as well as immunohistochemical detection of CD31, collagen (Col) I, Col III, CD3, CD68 and myeloperoxidase (MPO) were performed, as described previously in detail [15]. A BX53 microscope (Olympus, Hamburg, Germany) combined with the cellSens Dimension software (version 1.11; Olympus) was used for image acquisition and analysis. The density of CD31⁺ microvessels (expressed in mm^− 2^) was quantified in both the border and center zones of each implant by dividing the number of vessels by the corresponding ROI area. The proportion of GFP⁺ blood vessels (expressed as % of all vessels) was also determined for each group. In addition, the relative amounts of Col I and Col III were calculated in comparison to normal skin. Immune cell infiltration was evaluated by quantifying CD3⁺ lymphocytes, CD68⁺ macrophages and MPO⁺ neutrophilic granulocytes (expressed in mm^− 2^) in two central and two peripheral ROIs per implant.

### Statistical analysis

The size of the individual groups (*n* = 8) was determined based on experience from previous studies conducted in the same experimental setting [15]. All animals were included in the study. They were randomly assigned to the different groups. A blinded analysis was not performed. The normality and homogeneity of variance of all datasets were assessed using GraphPad Prism (version 10.1.2; GraphPad Software, San Diego, CA, USA). Depending on data distribution, group comparisons were conducted using either an unpaired Student’s t-test (parametric) or a Mann-Whitney rank-sum test (non-parametric). Results are expressed as mean ± standard error of the mean (SEM). Statistical significance was set at *p* < 0.05.

## Results

### Intravital fluorescence microscopy

**Fig. 2:**
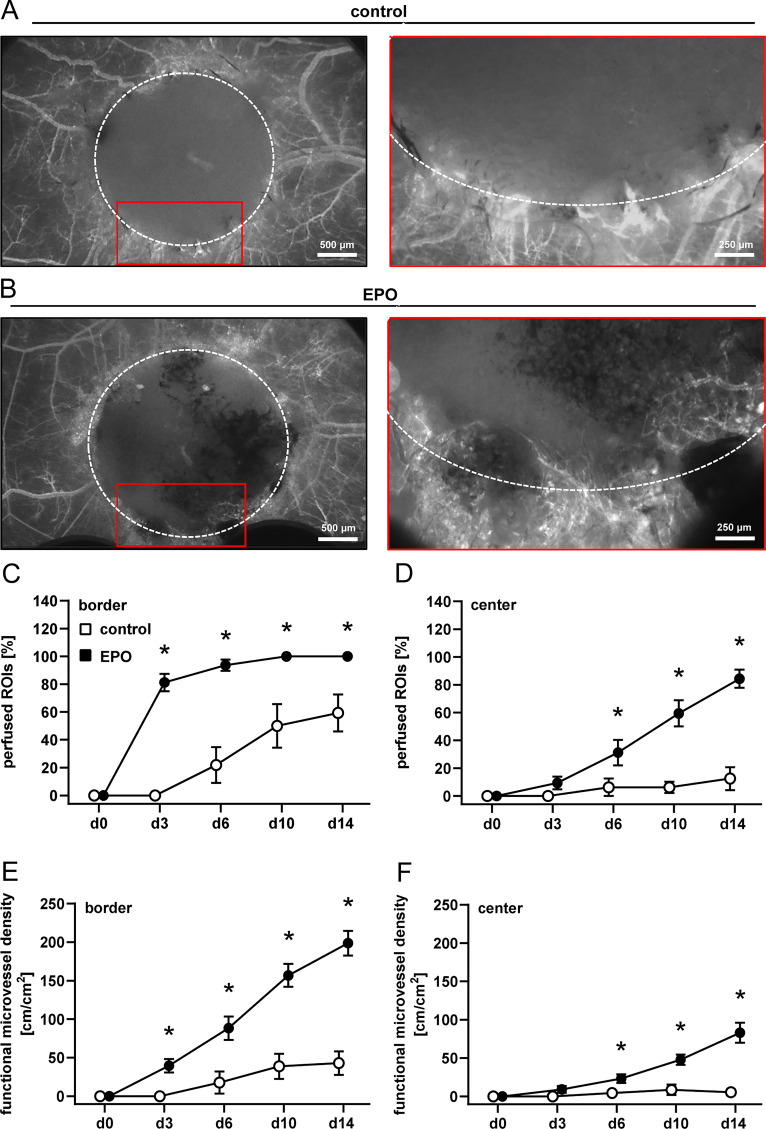
In vivo microscopy of nanofat-seeded dermal substitutes. (**A, B**) Representative intravital fluorescence microscopic images of dermal substitutes seeded with vehicle-pretreated nanofat (control, (**A**)) and EPO-pretreated nanofat (EPO, (**B**)) on day 14 after implantation into dorsal skinfold chambers of C57BL/6J recipient mice (broken line indicates implant borders; red frame highlights regions of interest (ROIs) at the implant border shown in higher magnification on the right). (**C-F**) Perfused ROIs (%) (**C, D**) and functional microvessel density (cm/cm²) (E, F) in the border (**C, E**) and center zones (**D, F**) of dermal substitutes seeded with vehicle-pretreated nanofat (control; white circles, *n* = 8) and EPO-pretreated nanofat (EPO; black circles, *n* = 8), as analyzed by intravital fluorescence microscopy on day (d) 0, 3, 6, 10 and 14 after implantation. Mean ± SEM. *p < 0.05 vs. control

Repeated intravital fluorescence microscopy enabled the detailed evaluation of the onset of blood perfusion and the progressive development of new microvascular networks in nanofat-seeded dermal substitutes that had been implanted into dorsal skinfold chambers of recipient mice. Notably, implants seeded with EPO-pretreated nanofat exhibited a much more pronounced microvascular ingrowth, both in the border and center zones over time, when compared to controls (Fig. 2A-F). This was reflected by a significantly higher number of perfused ROIs and increased functional microvessel density (Fig. 2C-F). Moreover, blood-perfused microvessels occurred significantly earlier at the borders of these implants already on day 3 after implantation. In contrast, dermal substitutes seeded with vehicle-pretreated nanofat exhibited a slower and less extensive development of novel and functional microvessels in their border and center zones (Fig. 2C-F). Microhemodynamic parameters of individual microvessels, such as diameter, centerline RBC velocity, shear rate and volumetric blood flow, were comparable in the two groups (Table 1). However, in the control group, these parameters could only be reliably assessed in the border zones of the implants between day 6 and 14, because the implants almost completely lacked blood-perfused microvessels at the other observation time points and in their central parts.

**Table 1:** Diameter (µm), centerline RBC velocity (µm/s), shear rate (s^− 1^) and volumetric blood flow (pL/s) of microvessels within the border and center zones of dermal substitutes seeded with vehicle-pretreated nanofat (control; *n* = 8) and EPO-pretreated nanofat (EPO; *n* = 8), as analyzed by intravital fluorescence microscopy on day (d) 0, 3, 6, 10 and 14 after implantation

	d0	d3	d6	d10	d14
**diameter (µm):**					
border: control	-	-	14.6 ± 0.0	15.4 ± 0.4	15.7 ± 1.0
EPO	-	12.2 ± 1.2	14.6 ± 0.9	14.2 ± 0.4	14.8 ± 0.3
center: control	-	-	-	-	-
EPO	-	9.0 ± 0.0	13.4 ± 1.7	13.4 ± 0.9	18.8 ± 3.6
***centerline RBC velocity (µm/s)***:					
border: control	-	-	158.8 ± 0.0	171.7 ± 21.7	191.1 ± 18.6
EPO	-	157.8 ± 29.3	163.6 ± 6.7	173.7 ± 9.4	197. 9 ± 3.8
center: control	-	-	-	-	-
EPO	-	93.2 ± 0.0	165.8 ± 6.8	202.1 ± 9.9	154.9 ± 23.2
***shear rate (s***^***-1***^***)***:					
border: control	-	-	89.2 ± 0.0	96.4 ± 14.5	103.7 ± 12.4
EPO	-	111.2 ± 0.0	97.1 ± 8.2	102.4 ± 7.6	111.4 ± 3.5
center: control	-	-	-	-	-
EPO	-	82.8 ± 0.0	108.6 ± 15.2	128.2 ± 11.9	84.7 ± 17.3
***volumetric blood flow (pL/s)***:					
border: control	-	-	16.7 ± 0.0	19.7 ± 2.6	24.5 ± 3.2
EPO	-	11.9 ± 4.1	18.1 ± 2.6	19.1 ± 1.5	21.8 ± 1.2
center: control	-	-	-	-	-
EPO	-	3.7 ± 0.0	14.7 ± 3.2	18.6 ± 2.4	23.3 ± 3.1

Mean ± SEM. No significant differences

**Fig. 3:**
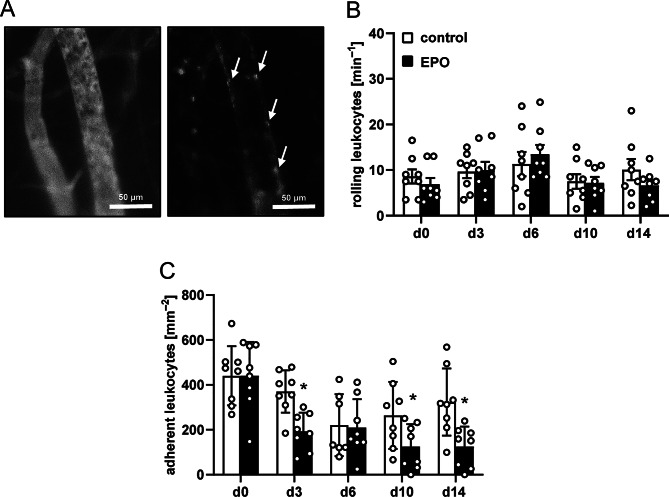
Leukocyte-endothelial cell interaction in response to nanofat-seeded dermal substitutes. (**A**) Representative intravital fluorescence microscopic images of a collecting venule next to a dermal substitute seeded with vehicle-pretreated nanofat (blue light epi-illumination, contrast enhanced by 5% FITC-labeled dextran; left panel) and green light epi-illumination with in situ staining of leukocytes using 0.1% rhodamine 6G (right panel) (arrows indicate leukocytes). (**B, C**) Rolling leukocytes (min^− 1^) (**B**) and adherent leukocytes (mm^− 2^) (**C**) within postcapillary and collecting venules next to dermal substitutes seeded with vehicle-pretreated nanofat (control; white bars, *n* = 8) and EPO-pretreated nanofat (EPO; black bars, *n* = 8), as analyzed by intravital fluorescence microscopy on day (d) 0, 3, 6, 10 and 14 after implantation. Mean ± SEM. *p < 0.05 vs. control

To evaluate the inflammatory response to the implants, leukocyte-endothelial cell interactions were examined in peri-implant postcapillary and collecting venules of the surrounding subcutaneous host tissue. For this purpose, leukocytes were labeled in situ with 0.1% rhodamine 6G for their visualization under green-light epi-illumination, while the microvessels were visualized under blue-light epi-illumination following injection of the plasma marker 5% FITC-labeled dextran (Fig. 3A). Of note, in both groups these microvessels did not differ in terms of diameter, centerline RBC velocity, shear rate and volumetric blood flow, indicating comparable microhemodynamic conditions (Table 2). However, while the number of rolling leukocytes was similar between the two groups (Fig. 3B), the number of adherent leukocytes was significantly lower on day 3, 10 and 14 in venules of the group of implants seeded with EPO-pretreated nanofat (Fig. 3C).

**Table 2:** Diameter (µm), centerline RBC velocity (µm/s), shear rate (s^− 1^) and volumetric blood flow (pL/s) of postcapillary and collecting venules in direct vicinity to dermal substitutes seeded with vehicle-pretreated nanofat (control; *n* = 8) and EPO-pretreated nanofat (EPO; *n* = 8), as analyzed by intravital fluorescence microscopy on day (d) 0, 3, 6, 10 and 14 after implantation

	d0	d3	d6	d10	d14
***diameter (µm):***					
control	34.5 ± 0.8	34.7 ± 0.8	33.8 ± 0.0	32.3 ± 0.9	32.3 ± 0.8
EPO	34.5 ± 0.8	37.6 ± 1.2	35.2 ± 0.9	32.6 ± 1.2	32.6 ± 0.9
***centerline RBC velocity (µm/s)*** *:*					
control	303.9 ± 31.5	309.6 ± 26.7	312.7 ± 40.5	241.4 ± 25.8	293.5 ± 27.4
EPO	288.6 ± 24.3	266.7 ± 17.9	245.8 ± 15.0	235.7 ± 21.8	263.2 ± 17.6
***shear rate (s***^***-1***^***)***:					
control	70.9 ± 7.1	71.4 ± 5.6	74.9 ± 8.7	60.0 ± 6.0	73.3 ± 6.6
EPO	67.4 ± 5.7	58.5 ± 4.8	56.1 ± 3.1	58.9 ± 6.0	65.6 ± 4.8
***volumetric blood flow (pL/s)***:					
control	187.6 ± 25.6	196.7 ± 23.8	184.2 ± 34.5	128.8 ± 20.5	157.0 ± 18.2
EPO	171.0 ± 17.2	192.3 ± 14.7	157.8 ± 16.2	127.4 ± 11.4	143.5 ± 12.6

Mean ± SEM. No significant differences

### Histology and immunohistochemistry

**Fig. 4:**
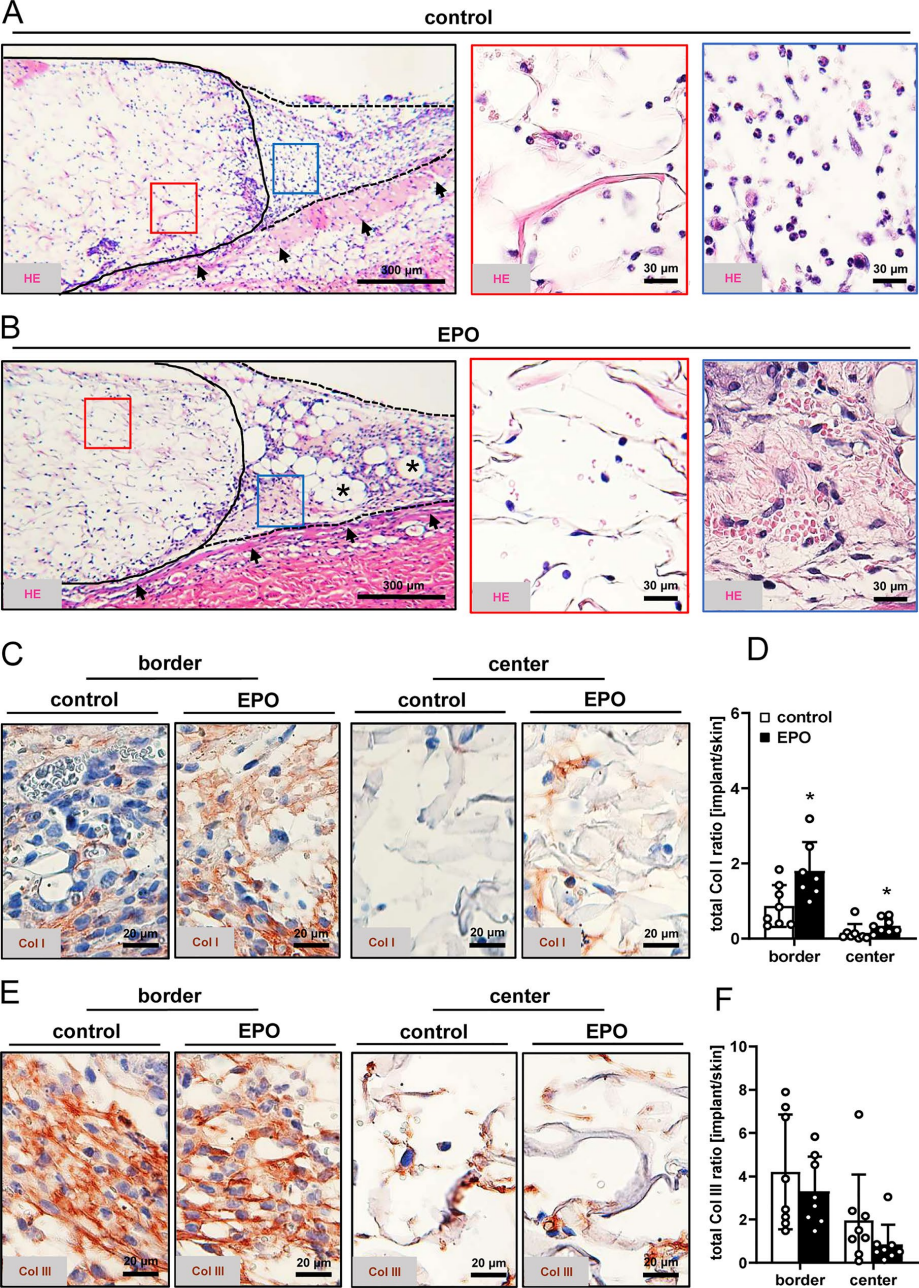
Tissue integration of nanofat-seeded dermal substitutes. (**A, B**) Representative HE-stained sections of dermal substitutes seeded with vehicle-pretreated nanofat (control, (**A**)) and EPO-pretreated nanofat (EPO, (**B**)) on day 14 after implantation into dorsal skinfold chambers of C57BL/6J recipient mice (closed line indicates implant border; broken line indicates border zone; blue and red frames indicate ROIs in the border and center zones of the implants shown in higher magnification on the left panels; arrows indicate panniculus carnosus muscle; asterisks indicate adipocytes). (**C, E**) Representative immunohistochemical sections showing Col I (**C**) and III (**E**) in the border and center zones of dermal substitutes seeded with vehicle-pretreated nanofat (control) or EPO-pretreated nanofat on day 14. (**D, F**) Total Col I (**D**) and Col III (F) ratio (implant/skin) in the border and center zones of dermal substitutes seeded with vehicle-pretreated nanofat (control; white bars, *n* = 8) and EPO-pretreated nanofat (EPO; black bars, *n* = 8) on day 14, as analyzed by immunohistochemistry. Mean ± SEM. *p < 0.05 vs. control

The implanted dermal substitutes were further examined by histology and immunohistochemistry at the end of the in vivo experiments on day 14. The analysis of HE-stained sections revealed an improved tissue integration of dermal substitutes seeded with EPO-pretreated nanofat when compared to controls (Fig. 4A, B). This was indicated by a more pronounced granulation tissue formation in the border zones of the implants. Additionally, these implants contained more residual adipocytes originating from the seeded nanofat (Fig. 4B). However, no evident differences were detected within the center zones of the implants between the two groups (Fig. 4A, B).

To further investigate tissue integration, the collagen content of the dermal substitutes was quantified, thereby distinguishing between Col I and Col III. This analysis revealed a significantly higher total Col I ratio in the border and center zones of dermal substitutes seeded with EPO-pretreated nanofat. In contrast, the overall Col III ratio did not differ between the two groups (Fig. 4C-F).

**Fig. 5:**
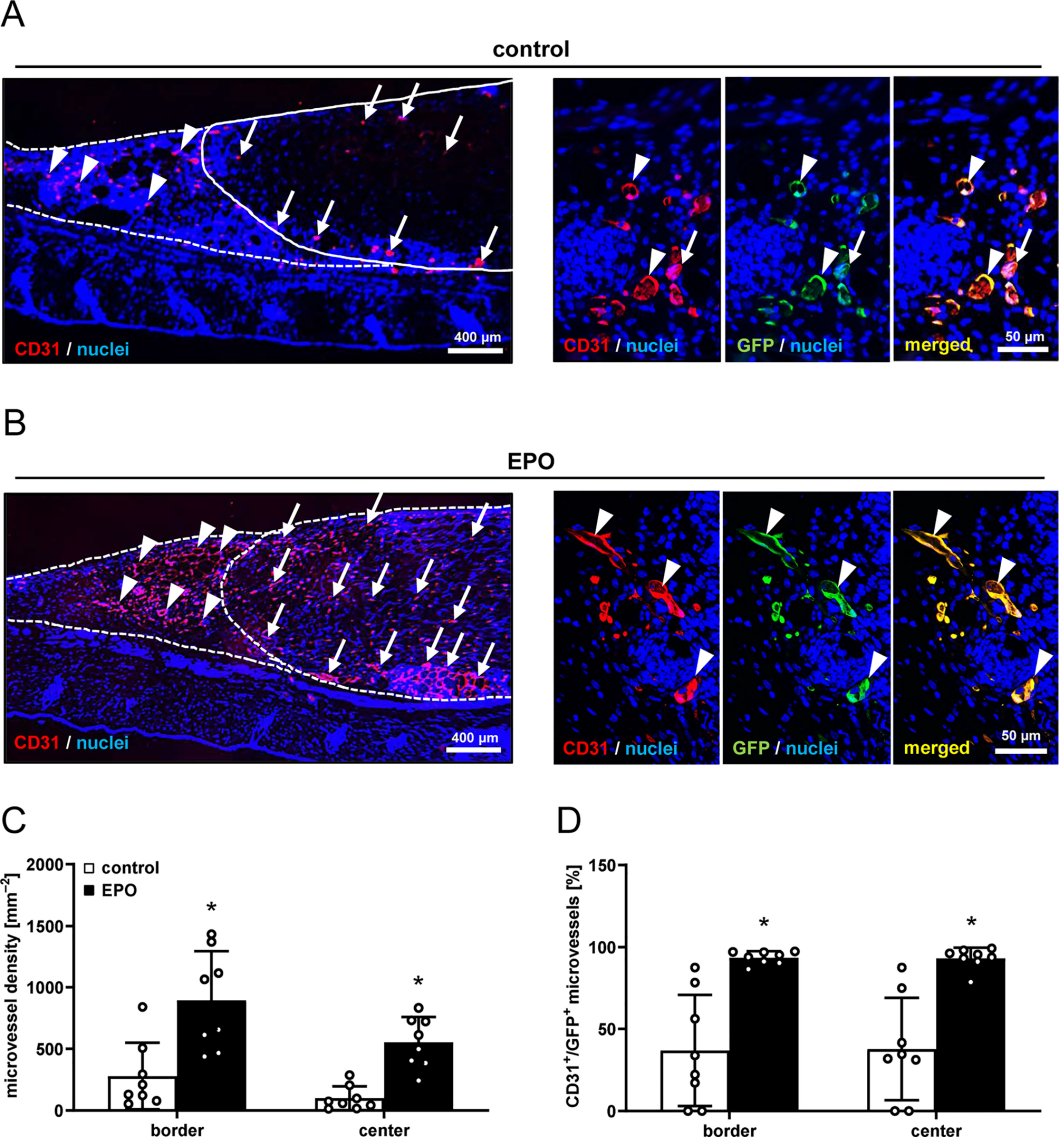
Vascularization of nanofat-seeded dermal substitutes. (**A, B**) Representative immunohistochemical sections showing CD31^+^ microvessels in the border zones (arrowheads) and center zones (arrows) as well as the detection of CD31^+^/GFP^−^ (arrows) and CD31^+^/GFP^+^ (arrowheads) microvessels of dermal substitutes seeded with vehicle-pretreated nanofat (control) or EPO-pretreated nanofat on day 14 after implantation into dorsal skinfold chambers of C57BL/6J recipient mice (closed line indicates implant border; broken line indicates border zones). (**C**) Microvessel density (mm^− 2^) of dermal substitutes seeded with vehicle-pretreated nanofat (control; white bars, *n* = 8) and EPO-pretreated nanofat (EPO; black bars, *n* = 8) on day 14, as analyzed by immunohistochemistry. (D) CD31^+^/GFP^+^ microvessels (%) in the border zones and center zones of dermal substitutes seeded with vehicle-pretreated nanofat (control; white bars, *n* = 8) and EPO-pretreated nanofat (EPO; black bars, *n* = 8) on day 14, as analyzed by immunohistochemistry. Mean ± SEM. *p < 0.05 vs. control

Additional immunohistochemical analyses were performed to detect CD31^+^ microvessels in the dermal substitutes. Implants seeded with EPO-pretreated nanofat exhibited a markedly higher functional microvessel density both in the border and center zones when compared to controls (Fig. 5A-C). Furthermore, CD31/GFP co-staining revealed that more than 90% of the microvessels within this group expressed GFP. This indicates that the newly formed vessels originated from the seeded GFP^+^ nanofat. In contrast, the fraction of GFP^+^ microvessels was markedly lower in dermal substitutes seeded with vehicle-pretreated nanofat (Fig. 5A, B, D).

**Fig. 6:**
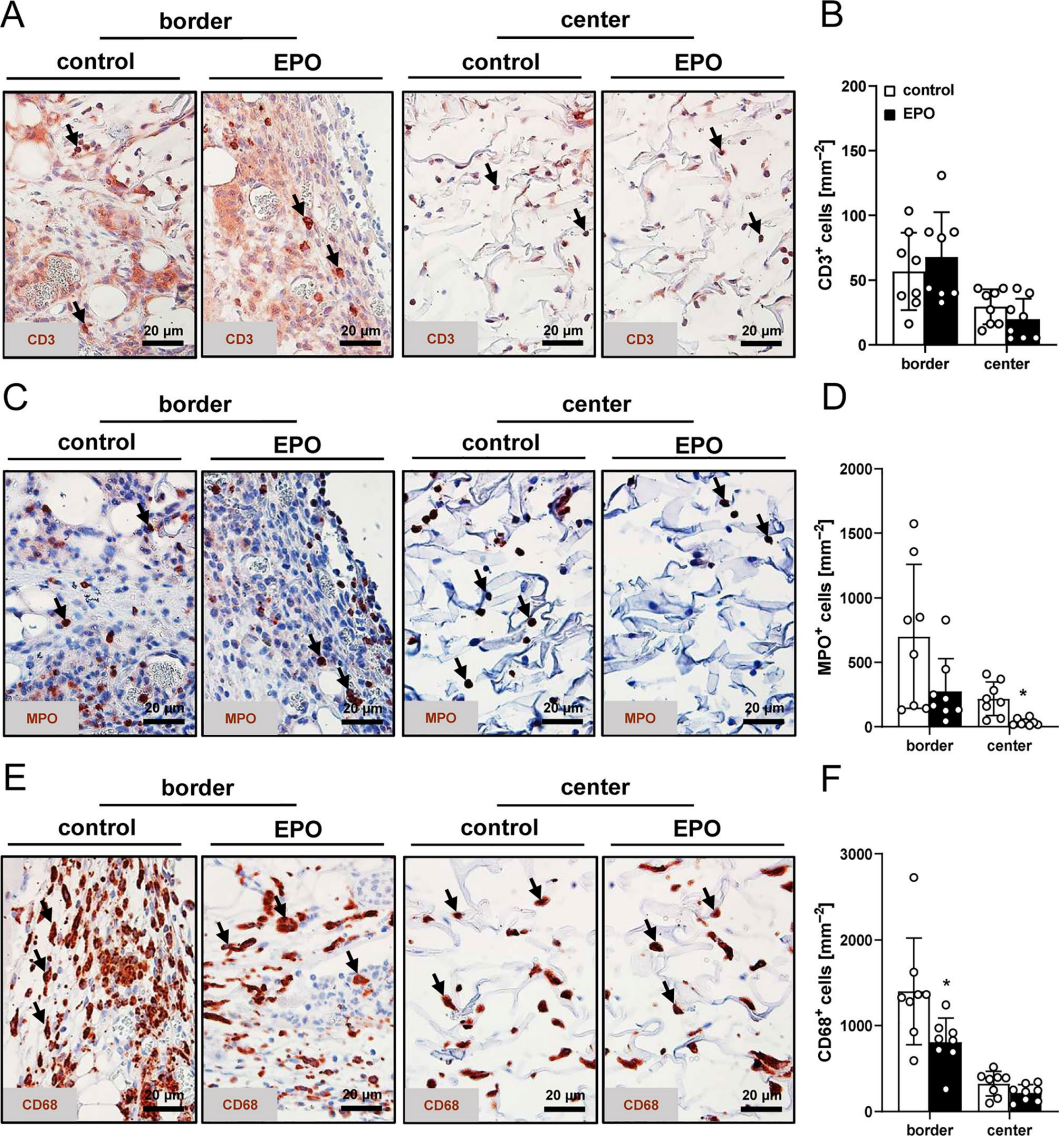
Infiltration of immune cells into nanofat-seeded dermal substitutes. (**A, C, E**) Representative immunohistochemical sections showing CD3^+^ lymphocytes (A, arrows), MPO^+^ granulocytes (C, arrows) and CD68^+^ macrophages (E, arrows) in the border and center zones of dermal substitutes seeded with vehicle-pretreated nanofat (control) and EPO-pretreated nanofat (EPO) on day 14 after implantation into dorsal skinfold chambers of C57BL/6J recipient mice. (B, D, F) CD3^+^ lymphocytes (mm^− 2^) (B), MPO^+^ granulocytes (mm^− 2^) (D) and CD68^+^ macrophages (mm^− 2^) (F) in the border and center zones of dermal substitutes seeded with vehicle-pretreated nanofat (control; white bars, *n* = 8) and EPO-pretreated nanofat (EPO; black bars, *n* = 8) on day 14, as analyzed by immunohistochemistry. Mean ± SEM. *p < 0.05 vs. control

Finally, histological sections were stained with antibodies against CD3^+^ lymphocytes, CD68^+^ macrophages and MPO^+^ neutrophilic granulocytes to assess the immune cell infiltration of the implants (Fig. 6A-F). Of interest, the quantitative analysis of these stained sections showed a lower density of CD68^+^ and MPO^+^ cells in implants seeded with EPO-pretreated nanofat (Fig. 6D, F), indicating an anti-inflammatory effect when compared to controls.

## Discussion

The regenerative capacity of nanofat can be further enhanced by exposure to bioactive stimuli, such as growth factors, platelet-rich fibrin, platelet-rich plasma, and glucose [17, 19, 23]. However, the effects of EPO on nanofat have not been investigated so far. The present study now demonstrates that short-term ex vivo exposure of nanofat to EPO markedly enhances its in vivo performance when seeded onto implanted dermal substitutes.

The vascularization capacity of nanofat was analyzed in the mouse dorsal skinfold chamber model by means of repeated intravital fluorescence microscopy. Dermal substitutes seeded with EPO-pretreated nanofat exhibited RBC-perfused microvessels as early as day 3 after implantation, whereas controls showed a delayed and spatially restricted perfusion. This early onset of vascularization was accompanied by a sustained increase in functional microvessel density over time, particularly within the central regions of the implants, which are typically most vulnerable to hypoxia [24]. These findings align with the well-known capacity of EPO to upregulate vascular endothelial growth factor (VEGF) expression, thereby promoting a pro-angiogenic environment. Moreover, through its receptor-dependent signaling pathways, including JAK2/STAT5 and PI3K/Akt, EPO enhances endothelial and progenitor cell survival and migration [2, 25]. In the context of nanofat, which is rich in endothelial cells and ASCs embedded within their native extracellular matrix, EPO exposure likely primes these cells to rapidly reassemble into functional microvascular networks and promotes their early angiogenic sprouting after implantation.

Notably, the vessels located in the peri-implant tissue as well as within the border and center zones of the implanted dermal substitutes did not exhibit alterations in microhemodynamic parameters, including vessel diameter, centerline RBC velocity, shear rate or volumetric blood flow, throughout the 14-day observation period. Accordingly, EPO pretreatment did not induce abnormal vessel remodeling or hyperperfusion, phenomena that are typically associated with increased microvessel diameters and dysregulated flow patterns [26]. These findings support the interpretation that EPO not only stimulates vascularization by increasing VEGF expression but also promotes vascular stabilization through direct actions on endothelial cells. In this context, the applied ex vivo dose of EPO was likely insufficient to trigger excessive nitric oxide (NO) production, which would otherwise lead to extensive vasodilation and increased blood flow. Such microhemodynamic NO-mediated alterations have been instead reported when higher and systemic EPO doses were administered (e.g., 5000 IU/kg) [2, 27].

Besides its pro-angiogenic effects, EPO also significantly modulated the inflammatory response to the implants. While leukocyte rolling in peri-implant venules was comparable between the experimental groups, the number of firmly adherent leukocytes was consistently reduced in the group of dermal substitutes seeded with EPO-pretreated nanofat. Because leukocyte adhesion represents a critical step preceding transendothelial migration into tissues, these findings indicate a selective attenuation of excessive inflammatory cell recruitment rather than a global suppression of immune surveillance [28]. This anti-inflammatory property of EPO may be attributed to its capacity to downregulate endothelial adhesion molecule expression, inhibit pro-inflammatory cytokine release and reduce oxidative stress [3]. In fact, systemic EPO administration has been proven to reduce the expression of tumor necrosis factor (TNF)-α and interleukin (IL)-1β in multiple models, including myocardial infarction [29], colitis [30] and hyperglycemia [31]. Hamed et al. [32] reported similar benefits after topical EPO administration in wounds of diabetic rats, primarily attenuating apoptotic cell accumulation and the subsequent inflammatory signaling cascade. In the current setting, these EPO effects likely contributed to a more permissive microenvironment for early vascular ingrowth and tissue integration of the nanofat-seeded dermal substitutes.

In line with this view, histological analyses on day 14 showed an improved tissue integration of dermal substitutes seeded with EPO-pretreated nanofat. Enhanced granulation tissue formation at the implant borders together with a higher number of residual adipocytes indicated a better survival of the transplanted adipose tissue components. This observation is in line with previous studies showing that EPO protects adipocytes and stromal cells from apoptosis and ischemic injury, thereby preserving the structural and paracrine integrity of adipose grafts [33, 34]. The preservation of viable adipose tissue within the dermal substitutes may have further supported angiogenesis through a sustained release of angiogenic and trophic factors. Moreover, immunohistochemical analyses on day 14 revealed a significant increase in Col I deposition in both border and center zones of implants seeded with EPO-pretreated nanofat, whereas Col III levels remained unchanged. Col I is a hallmark of mature, mechanically stable connective tissue, while collagen III predominates in early granulation tissue [35]. Thus, the observed selective increase in Col I suggests that EPO pretreatment may have accelerated matrix maturation rather than merely amplifying fibrotic responses. This effect may be indirectly caused by improved vascularization and reduced inflammation, as adequate perfusion and controlled immune activation are key determinants of balanced extracellular matrix remodeling [36].

Immunohistochemical detection of CD31⁺ microvessels further demonstrated that EPO pretreatment significantly increases the microvessel density within the implants. Notably, more than 90% of these vessels were GFP⁺, indicating their origin from the seeded nanofat. On the other hand, only 37% of vessels in the control group were GFP⁺. These findings underscore the central function of EPO in promoting nanofat-dependent vascularization over host tissue-driven angiogenesis. Similar observations were reported for MVFs pretreated with EPO, where enhanced endothelial proliferation and survival translated into improved in vivo network formation [5]. Such a mechanism may be especially beneficial in clinical situations characterized by an impaired angiogenic activity of the treated host site, e.g. following radiation exposure or extensive traumatic injuries, such as burns [37, 40].

The present study further illustrates the enhanced biocompatibility of dermal substitutes seeded with EPO-pretreated nanofat. The significantly reduced density of CD68⁺ macrophages and MPO⁺ neutrophilic granulocytes within EPO-treated implants provides further evidence for an improved inflammatory microenvironment. In fact, excessive infiltration of these innate immune cells is well known to compromise vascular stability and tissue integration through the release of proteolytic enzymes and reactive oxygen species [41, 42]. In line with our findings, Hamed et al. [33] reported a marked reduction in CD68⁺ macrophage accumulation in EPO-treated fat grafts following subcutaneous implantation in mice, which was associated with decreased apoptotic cell death. Moreover, accumulating evidence indicates that EPO actively modulates innate immune responses by promoting macrophage polarization toward a pro-regenerative phenotype while simultaneously limiting neutrophil-mediated tissue injury [3]. Collectively, these immunomodulatory effects likely contributed to the improved vascular integrity and tissue integration observed in EPO-pretreated nanofat-seeded dermal substitutes.

The choice of EPO concentration and exposure duration represents a critical aspect in EPO therapy. EPO exerts most of its properties in a dose-dependent manner [29, 43]. Accordingly, higher dosages of this hormone may cause hypertension and microvascular thrombosis when given systemically [4, 44]. Therefore, in the present study, a relatively low concentration of 3 IU/mL EPO was selected for a 1-hour ex vivo pretreatment of nanofat. This dosage and type of treatment were able to achieve sufficient cellular activation while minimizing the risk of adverse effects in vivo [45]. Moreover, the relatively short exposure time in the present study supports a potential clinical translation of this approach in the future, as autologous nanofat is typically processed and immediately reapplied intraoperatively.

Finally, some limitations of the present study should be considered. The experiments were conducted in a mouse dorsal skinfold chamber model, which allows high-resolution analysis of vascularization but does not fully recapitulate the complexity of clinical wound environments. Furthermore, this approach only enables the assessment of implant vascularization during the first 14 days. Although this initial period may be particularly relevant for dermal substitutes, it would be interesting to study the long-term durability, stability and function of the nanofat-seeded implants, which may be performed in clinically relevant large animal models in the future. In such models, it may be also better possible to assess additional parameters, such as long-term mechanical strength, barrier function and wound closure capacity of the implants. In addition, only a single EPO dose and exposure duration were investigated in the present study. Future studies should therefore also address dose- or time-response relationships.

## Conclusions

This study shows that short-term ex vivo pretreatment of nanofat with EPO is an effective and clinically translatable strategy to enhance vascularization, improve tissue integration and modulate inflammation in nanofat-seeded dermal substitutes. By accelerating the establishment of a functional microvascular network without compromising vessel quality or biocompatibility, this approach addresses a key limitation of dermal substitutes and holds promise for improving their future performance in reconstructive surgery and complex wound management.

## Data Availability

All data generated or analyzed during this study are included in this published article.

## Abbreviations

ASC: Adipose-derived stem cell
Col: Collagen
EPO: Erythropoietin
FITC: Fluorescein isothiocyanate
GFP: Green fluorescent protein
HBSS: Hank’s Balanced Salt Solution
HE: Hematoxylin and eosin
IL: Interleukin
IU: International unit
JAK: Janus kinase
MPO: Myeloperoxidase
MVF: Microvascular fragment
NIH: National Institutes of Health
NO: Nitric oxide
PI3K: Phosphoinositide-3-kinase
RBC: Red blood cell
ROI: Region of interest
SEM: Standard error of the mean
STAT: Signal transducer and activator of transcription
TNF: Tumor necrosis factor
VEGF: Vascular endothelial growth factor
